# Chronic Anterior Pituitary Dysfunction Following Traumatic Head Injury: Prospective Study in Hospital Sultanah Aminah Johor Bahru, Malaysia

**DOI:** 10.21315/mjms2023.30.1.9

**Published:** 2023-02-28

**Authors:** Nurshaheda Mohd Salleh, Sharon Casilda Theophilus, Noor Azman A Rahman, Abdul Rahman Izaini Ghani, Jafri Malin Abdullah, Zamzuri Idris, Zi Han Tan, Nur Muhammad Kamil

**Affiliations:** 1 Department of Neurosurgery, Hospital Sultanah Aminah, Johor, Malaysia; 2 Department of Neurosciences, School of Medical Sciences, Universiti Sains Malaysia, Kelantan, Malaysia; 3 Brain and Behaviour Cluster, School of Medical Sciences, Universiti Sains Malaysia, Kelantan, Malaysia

**Keywords:** chronic anterior pituitary dysfunction, traumatic brain injury, quality of life, SF-36

## Abstract

**Background:**

Hypopituitarism following traumatic brain injury (TBI) is not rare however most patients were left undiagnosed and untreated. Association of post TBI hypopituitarism causing neurobehavioural and quality of life impairment. The aim of the study is to determine the incidence of the chronic anterior pituitary deficiency in patients with traumatic brain injury. Subsequently determine the risk factor and the outcome of the patient with chronic anterior pituitary dysfunction.

**Methods:**

This is single centre cross-sectional study involved 105 traumatic head injury patients under the Neurosurgical Department Hospital Sultanah Aminah, Johor Bahru, Malaysia. The primary investigator will do an interview and the patients will be asked question to complete a questioner from SF-36 (36 questions). Subsequently, consent for participation will be taken and blood sampling will be done.

**Results:**

Thirty-three patients were noted to have anterior pituitary dysfunction. The mean age was 36.97 ± 12.96 years old. Twenty-seven patients (32.5%) were male and six patients were female (27.3%). Chronic anterior pituitary dysfunction in patients with a severe traumatic head injury around 47.1% (23 patients), as compared to a moderate head injury (8 patients, 38.1%) and 2 sustained mild head injury (5.6%). The mean duration after the onset of trauma was 10.3 ± 1.79 months. All patient with anterior pituitary dysfunction had positive CT brain findings with 22 had subarachnoid haemorrhage (SAH) at the basal cistern and 27 patients had a base of skull fracture, where 52.1% of the patient underwent surgical intervention, 84.8% involved one axis and another 5 patients had two axes involved. Severity of the head injury (*P* < 0.001), prolonged duration of hospital stay (*P* = 0.014), radiological findings of a base of skull fracture (*P* < 0.001) and presence of SAH at basal cistern (*P* < 0.001) was significantly associated with pituitary dysfunction. The patient with anterior pituitary dysfunction has the lower 36-item Short Form Survey (SF-36) marks 56.3 ± 10.3.

**Conclusion:**

The prevalence of hypopituitarism was 31%. Indicators are increased TBI severity, prolonged hospitalisation and positive finding in radiological assessment. Post-traumatic chronic anterior pituitary dysfunction also related with poor quality of life as showed by low SF-36 marks.

## Introduction

Traumatic brain injury (TBI) is a significant cause of death and disability in young adults, with the added complication of physical disabilities leading to long-term cognitive, behavioural, psychological and social defects. Based on a census of head injuries conducted by the Neurosurgical Department of the Hospital Sultanah Aminah, about 998 and 982 cases of head injury were reported for the adult population in Johor 2016 and 2017, respectively. Out of the 998 cases of head injury in 2016, severe head injury comprised 335 cases and moderate head injury comprised 102 cases. For 2017, there were 324 cases of severe head injury and 98 cases of moderate head injury.

Hypopituitarism is defined as the total or partial loss of anterior and posterior pituitary gland function caused by pituitary or hypothalamic disorders. Hypopituitarism can be divided into primary or secondary forms. TBI is a common cause of secondary hypopituitarism, ranging from 30%–70% (1).

Post-traumatic endocrine dysfunction has been reported as a complication of TBI, which causes serious physical and mental effects in patients. One study of note reported that TBI-induced hypothalamic-pituitary dysfunction may contribute to poor quality of life in many TBI survivors (2). Several recent cohort studies have indicated that 25%–40% of TBI patients will develop chronic hormonal deficiencies (2). One study in particular showed that the prevalence of anterior pituitary dysfunction was 30%–70%, with the somatotroph and gonadotroph axes commonly affected, and the thyrotrophin and corticotrophin axes being less affected (3). Furthermore, a prospective study showed that 50.9% of patients with TBI had at least one anterior pituitary hormone deficiency after 1 year (3).

Hormonal evaluations performed in the early stages of TBI (i.e. a mean of 12 days after trauma) revealed a substantial prevalence of pituitary deficits (4). However, it is unclear at this time whether these hormonal changes are specifically due to brain trauma or whether they are an unspecific reaction to critical illness. After TBI, the frequency of chronic hypopituitarism (usually defined as lasting at least 3 months) varies substantially between studies, with most indicating a range of 15%–50% (4).

The pathophysiology of anterior pituitary hormone deficiency following TBI has indicated that a role is played by both direct mechanical injury and injury from hypotension, hypoxia, anaemia and brain swelling, causing a restriction of flow in the long hypophyseal portal vessels (3). The clinical symptoms depend on the severity of hypopituitarism and the number of deficient anterior pituitary hormones. The clinical presentation seen in cases of hypopituitarism varies from very subtle findings to life-threatening conditions in which patients are attended to in the emergency department because of severe manifestations of pituitary failure, such as adrenal crisis, severe hypotension, hypoglycemia and hypothyroidism. Due to the subtle manifestations of the condition, most patients with hypopituitarism remain undiagnosed and are therefore untreated. Among the clinical radiological parameters, diffuse axonal injury and basal skull fracture were found to be associated with an increased prevalence of TBI-induced hypopituitarism (4).

The Endocrine Society Clinical Practice Guidelines include recommendations for the diagnosis of hypopituitarism. Specifically, the guidelines recommend measuring serum cortisol levels from 8:00 a.m. to 9:00 a.m. as a test for diagnosing central adrenal insufficiency (AI). A cortisol level < 3 μg/dL is indicative of AI. For central hypothyroidism, the normal free T4 level would be 12.0 nmol/L–22.0 nmol/L. Any level below the laboratory reference range is considered indicative of hypothyroidism. Thyroid stimulating hormone (TSH) in the setting of pituitary disease is usually confirmatory. In the absence of acute/subacute illness, blood testosterone, follicular stimulating hormone FSH) and luteinizing hormone (LH) levels, as well as serum prolactin (PRL) levels, should be measured in males with suspected hypogonadism. Serum estradiol (E2), FSH and LH should all be measured in the presence of oligomenorrhea or amenorrhea. Clinicians should rule out alternative reasons for menstrual irregularities that are not connected to ovulation problems. The absence of elevated serum FSH and LH in post-menopausal women is sufficient for a diagnosis of gonadotrope malfunction (assuming the patient is not on hormone replacement treatment) (4).

The 36-Item Short Form Survey (SF-36) is a popular instrument for evaluating health-related quality of life (QoL). The SF-36 was developed in the USA for use in the Rand Corporation’s Health Insurance Experiment (5) and has been shown to yield reliable scores on scales measuring eight dimensions of health status, as well as on summary measures of physical and mental health components. The SF-36 includes eight scales that measure physical functioning (PF), role physical (RP), bodily pain (BP), general health (GH), vitality (VT), social functioning (SF), role emotional (RE) and mental health (MH). The SF-36 has been used to examine the QoL of patients after TBI, and it was found that all dimensions were affected and caused poor quality of life in these patients (6).

This study aims to evaluate the incidence of chronic anterior pituitary deficiency in patients with TBI in the Neurosurgical Department of Sultanah Aminah Hospital in Johor Bahru, to identify the predictive factors that increase the risk of chronic anterior pituitary deficiency after TBI and to assess QoL in patients with chronic anterior pituitary dysfunction.

## Methods

### Study Design and Population

This was a prospective cohort study conducted at the Sultanah Aminah Hospital in Johor Bahru from October 2019 to October 2020. All patients with traumatic head injuries who were admitted to Hospital Sultanah Aminah, Johor Bahru and fulfilled the inclusion criteria were enrolled in the study. The inclusion criteria were patients with traumatic head injury, an age between 20 years old and 60 years old, and a history of head injury 3 months prior to enrollment in the study. Patients who had traumatic head injury and were in a chronic vegetative state with low life expectancy, those were diagnosed or taking hormonal treatment prior to the trauma and pregnant women were excluded from the study.

### Method of Research

Selected patients were given a research identification number after consenting to participate in the study. Patients with good cognitive function who understood Malay or English were asked to complete the SF-36 questionnaire on their own. The estimated time to complete the questionnaire was 10 min– 20 min. If they were unable to complete it, the primary investigator explained the question(s) to the respondent or caretaker and completed the form based on the answer(s) given. Subsequently, patients were given an appointment date for blood tests at the clinic within two weeks’ time. The results from the questionnaire were converted to a 100-point scale via a calculator provided.

Patients were required to fast prior to the appointment and samples were taken between 8.00 a.m. and 9.00 a.m. in the morning. A total of 10 cc of blood was taken from each patient and kept in two sodium heparinised bottles and one plain tube. Each sample was to be tested for serum cortisol, LH, FSH, T4 and TSH levels and sent to the biochemistry lab to be processed. Any investigation of growth hormone levels was excluded from this study. The results of the hormonal level testing were reviewed by the primary investigator and under guidance of an endocrinologist. The patients’ admission records were traced and the clinical data were consulted. Radiological imaging was recorded based on an official radiologist report.

### Statistical Analysis

The data analysis was performed using SPSS version 26.0 statistical software. Descriptive data were expressed as means ± standard deviations (SD) unless otherwise stated. Frequency tests were conducted to determine the incidence of chronic anterior pituitary deficiency in patients with TBI and the outcome (QoL). Other parameters, such as age, gender, severity of the traumatic head injury, length of stay, duration post-trauma, mode of treatment and radiological findings, were subjected to a binary logistic regression analysis to determine the relationship between the parameters and chronic anterior pituitary deficiency.

## Results

### Demographic and Clinical Characteristics

A total of 105 patients who sustained traumatic head injuries and fulfilled the inclusion criteria were enrolled in the study. The clinical characteristics of the patients are summarised in Table 1. Of the 105 patients, 83 (79%) were male and 22 (21%) were female. The age distribution of patients showed that 62.9% were young adults (18 years old–40 years old) and 39% were middle-aged adults (41 years old–60 years old). The mean age was 36.91 ± 12.42 years old. Each patient was enrolled for an average of 9.88 ± 2.29 months after the trauma, with the minimum duration being 6 months, and the maximum duration 15 months. A total of 48 (45.7%) patients sustained severe head injuries, 21 (20%) patients had moderate head injuries and 36 (34.4%) patients had mild head injuries. The percentage of patients with positive CT brain findings was 58.1% (61 patients), while 44% had negative findings. While 48 patients underwent surgical intervention, 22 patients (21%) underwent decompressive craniectomy, 21 patients (20%) had craniotomies and 7% had intracranial pressure probes inserted. Around 53 (50%) of the patients had hospital stays between 1 and 14 days, and only five patients (4.8%) had prolonged hospital stays for more than 30 days. We also observed that the mean score of the SF-36 was 70 ± 13.13, with the highest score being 90 and the lowest being 30. Of this group of patients, 33 (31.4%) developed chronic anterior pituitary dysfunction and 72 (68.6%) had no abnormalities.

### Chronic Anterior Pituitary Dysfunction

Table 2 shows the clinical comparison between patients with anterior pituitary dysfunction and those without the condition. A total of 33 patients were noted to have anterior pituitary dysfunction. The mean age was 36.97 ± 12.96 years old, with a median age of 35 years old. Out of these 33 patients, 27 (32.5%) were male and six were female (27.3%). The difference in gender was not considered a risk factor for developing chronic anterior pituitary dysfunction (*P* = 0.637). Chronic anterior pituitary dysfunction was also seen in 47.1% of patients with a severe traumatic head injury; a total of 23 patients sustained a severe head injury as compared to a moderate head injury (8 patients, 38.1%), and two sustained mild head injuries (5.6%).

The mean duration after the onset of trauma was 10.3 ± 1.79 months and the median duration was 11 months. All patients with anterior pituitary dysfunction had abnormal CT brain findings. Out of these 33 patients, 22 had subarachnoid haemorrhage (SAH) at the basal cistern and 27 patients had a base of skull fracture; 52.1% of the patients underwent surgical intervention, while 8 did not. The most frequent procedure performed was decompressive craniectomy, followed by craniotomy and intracranial pressure (ICP) probe insertion.

Univariate logistic regression analysis (Table 3) was used to examine the relationship between each of the variables and showed that the severity of the head injury (*P* < 0.001), prolonged duration of hospital stay (*P* = 0.014), radiological findings of a base of skull fracture (*P* < 0.001) and presence of SAH at the basal cistern (*P* < 0.001) were significantly associated with chronic anterior pituitary dysfunction. A total of 23 of the patients had a base of skull fracture and another 31 had SAH present in their basal cistern. The odds ratio of developing anterior pituitary dysfunction if the patient had a base of skull fracture was 4.32 (95% CI: 1.781, 10.497). The odds ratio for patients with subarachnoid haemorrhage at the basal cistern was 3.33 (95% CI: 1.401, 7.932). We also observed that patients with pituitary dysfunction had prolonged stays in the hospital (84.8%) compared to patients without the condition. There was also a significant difference in the SF-36 scores, where the patients with anterior pituitary dysfunction had a mean SF-36 score of 56.3 **±** 10.3 as compared to those with no pituitary dysfunction scoring 77.74 ± 7.73.

Out of all 33 patients with chronic anterior pituitary dysfunction, 84.8% of the cases involved one axis, and another five patients had two axes involved. Table 4 shows that the most affected hormone by head trauma was the gonadotroph axis (54.5%), followed by the thyrotrophin (42.4%) and corticotrophin (18.2%) axes. A single hormonal defect was the most prevalent abnormality in 28 patients, followed by two axes in five patients, but no patient was diagnosed with three-axis dysfunction.

## Discussion

TBI is defined as a change in brain function or other evidence of brain pathology caused by external forces and the condition is a well-recognised public health problem worldwide. In Malaysia, head injury was found to be the fifth (7.86%) most common cause of hospitalisation. As a major referral and tertiary neurosurgical centre, Hospital Sultanah Aminah Johor Bahru serves as the largest referral centre for traumatic head injury cases in the entire state of Johor and the southern region of peninsular Malaysia. This was a prospective study conducted in Southeast Asia to determine the prevalence of chronic anterior pituitary dysfunction and its outcomes. A total of 105 patients were enrolled in the study, with a higher proportion of males in relation to females.

A total of 31% of our patients had developed certain pituitary dysfunction, which is similar to a previous study indicating a range between 15% and 60%. A multicentred study showed that 14.1% of their patients had been diagnosed with post-traumatic hypopituitarism (7). Another study showed that 60% of their 28 patients had pituitary dysfunction (8). Our study had comparable results with those of previous studies with similar inclusion and exclusion criteria.

A single hormonal deficit was found to be more prevalent than multiple hormonal abnormalities. This finding is in concordance with another study, which also found that almost 51% of patients had a single hormonal deficit compared with 17% having a combined deficit. Most investigations have found that GH deficiency is the most prevalent pituitary endocrine defect after TBI, with rates of 44% (4) and 18.6% (9) being reported; followed by the gonadal axis deficient (15%) then the thyrotrophin axis (10). While the present study did not include growth hormone, we found the gonadal axis to be the most common axis affected, followed by thyrotropin and corticotrophin.

The development of post-traumatic hypopituitarism is related to the primary mechanical insults of the sella turcica or secondary insults. The aim of this study was to determine the risk factors for developing anterior pituitary dysfunction and the outcomes of patients with chronic pituitary dysfunction. The severity of the head injury is one of the risk factors for developing pituitary dysfunction. In Schneider’s series, the prevalence of pituitary dysfunction in cases of severe, moderate, and mild TBI were 35.3%, 10.9% and 16.8%, respectively (11). Our study showed a slightly higher prevalence compared to the case series at 47.1%. This is likely due to the high incidence of severe head injuries in our study. The severity of the head injury is related to the radiological findings of brain swelling and midline shift, which supported the pathophysiology of developing hypopituitarism post-traumatic head injury.

Yang *et al*. (12) found that basal skull fractures were more significantly associated with pituitary dysfunction than ICH. Similarly, our study showed that the base of skull fracture was a risk factor for developing pituitary dysfunction. We also found that having a subarachnoid bleed (SAH) at the basal cistern raised the risk of developing anterior pituitary dysfunction (70.5%). A study by Kelly et al. (13) showed that 40% of patients who had SAH at the cistern developed pituitary dysfunction. Multiple studies have also shown that prolonged hospital stay is one of the contributing factors of pituitary dysfunction. A study by Yaseen et al. (8) showed an increased prevalence of pituitary dysfunction for patients with prolonged ICU stays, but this was not studied in our paper.

In individuals with moderate-to-severe TBI, almost 70% attained good functional recovery at early follow-up (3 months) but 30% still had a poor functional outcome (2). However, patients with pituitary hypopituitarism showed a marked reduction in QoL as compared to the traumatic head injury population without hypopituitarism. This is consistent with the findings of other studies, such as the one conducted by Andelic et al. (6), who also used the SF-36 to examine the QoL of patients after TBI; in this case, they found that all dimensions were affected in their patients.

## Conclusion

The prevalence of hypopituitarism was estimated to be 31% in the patients included in the present study. Indicators of the condition were increased TBI severity, prolonged hospitalisation and positive findings in radiological assessment (i.e. presence of SAH and base of skull fracture). Post-traumatic chronic anterior pituitary dysfunction was also associated with poor QoL, as shown by low SF-36 scores. Despite the SF-36 score not being a specific measurement tool for post-traumatic hypopituitarism, it can be used as a screening tool prior to more invasive screening approaches, such as blood taking. Therefore, early detection and intervention should be prioritised to improve the QoL of post-traumatic patients.

## Figures and Tables

**Table 1:** Clinical characteristics of patients

		*N*	%
Age, mean (SD)		36.91 (12.42) years old	
Duration after trauma, mean (SD)		9.8 (2.2) months	
Gender	Male	83	79.0
	Female	22	21.0
Classification of head injury	Mild	36	34.3
	Moderate	21	20.0
	Severe	48	45.7
CT brain findings	Negative	44	41.9
	Positive	61	58.1
Duration of hospital stay (days)	1–14	53	50.5
	15–30	47	44.8
	>30	5	4.8
Surgical intervention	No intervention	57	54.3
	Intervention	48	45.7
Type of surgical intervention	Decompressive craniectomy	22	21.0
	Craniotomy	21	20.0
	Intracranial pressure monitoring	7	6.7
	No intervention	55	52.4
SF-36 score	0–20	0	0
	21–40	1	1.0
	41–60	24	22.9
	61–80	49	46.7
	80–100	31	29.5

**Table 2:** Comparison of chronic anterior pituitary dysfunction patients with the rest of the cohort

		Yes *n* (%)	No *n* (%)	*P*-value
Total		33 (31.4)	72 (68.6)	
Age		36.97 (± 12.96)	36.89 (± 12.27)	
Duration post-trauma		10 (± 1.89) months	9.76 (± 2.46) months	
Gender	Male	27 (32.5)	56 (67.5)	0.633
	Female	6 (27.3)	16 (72.7)	
Classification of head injury	Mild	2 (5.6)	34 (94.4)	0.462
	Moderate	8 (38.1)	13 (61.9)	< 0.001^a^
	Severe	23 (47.1)	25 (52.1)	< 0.001^a^
CT brain findings				
Base of skull fracture	Present	23 (47.7)	25 (52.3)	< 0.001^a^
	Absent	10 (16.4)	51 (83.6)	
SAH at basal cistern	Present	31 (70.5)	13 (29.5)	< 0.001^a^
	Absent	2 (3.3)	59 (96.7)	
Duration of hospital stay (days)	1–14	13 (24.5)	40 (75.5)	0.923
	15–30	15 (31.9)	32 (68.1)	0.124
	> 30	5 (100)	0 (0)	0.002^a^
Surgical intervention	No intervention	8 (14.0)	49 (86.0)	0.878
	Intervention	25 (52.1)	23 (47.9)	
SF-36 score mean (SD)		56.3 ± 10.322	77.74 ± 7.730	

Notes:

a Pearson’s chi-square test: significant at *P* < 0.05

**Table 3:** Multiple logistic regression analysis of the factors affecting dysfunction chronic pituitary

		B (SE)	Adjusted odds ratio (95% CI)	*P*-value
Age (years old)				
18–40		−0.326 (0.431)	1.385 (0.596, 3.221)	0.448
41–60				
Duration post-trauma		10 (± 1.89)^a^ months	9.76 (± 2.46)^a^ months	0.852
Gender	Male	−0.780 (−0.210)	0.778 (0.274–2.211)	0.637
	Female			
Classification of head injury	(Constant)			
	Mild (C)	−2.462 (0.933)	0.907 (0.14–0.531)	< 0.001
	Mild 1	−2.332 (0.887)	0.887 (0.15–0.443)	< 0.001
	Mild 2	−2.345 (0.667)	0.776 (0.13–0.233)	< 0.001
(Mid)	Moderate	−2.445 (0.443)	0.445 (0.23–0.435)	0.344
	Moderate 1	−2.567 (0.734)	0.873 (0.14–0.341)	0.564
	Moderate 2	−1.532 (0.054)	0.773 (0.12–0.651)	0.421
(Mid)	Severe	−2.334 (0.998)	0.567 (0.23–0.452)	0.642
	Severe 1	−2.347 (0.321)	0.432 (0.11–0.321)	0.543
	Severe 2	−1.654 (0.021)	0.332 (0.12–0.421)	0.211
CT brain findings				
Base of skull fracture	Present	0.780 (0.210)	4.324 (1.781, 10.497)	< 0.001^a^
	Absent			
SAH at basal cistern	Present	1.204 (0.442)	3.333 (1.40, 7.932)	0.005
	Absent			
Duration of hospital stay (days)	1–14	0.917 (0.372)	2.501 (1.207–5.184)	0.014
	15–30			
Surgical intervention	No intervention	−1.89 (0.478)	6.658 (2.607–17.004)	0.878
	Intervention			
SF-36 score		56.3 ± 10.322	77.74 ± 7.730^b^	0.756

Notes: SE = standard error; CI = confidence interval; B = logistic regression coefficient

**Table 4:** Criteria for chronic anterior pituitary dysfunction

Chronic anterior pituitary dysfunction		*N* = 33	31.4%
Axis involved	1 axis	28	84.8%
	2 axis	5	15.2%
	3 axis	0	0
Gonadotrophin	Normal	15	45.5%
	Abnormal	18	54.5%
Thyrotrophin	Normal	19	57.6%
	Abnormal	14	42.4%
Corticotrophin	Normal	27	81.8%
	Abnormal	6	18.2%
SF-36 (score)		56.3 ± 10.322	
